# Assessing the Relationship Between High-sensitivity C-reactive Protein and Kidney Function Employing Mendelian Randomization in the Japanese Community-based J-MICC Study

**DOI:** 10.2188/jea.JE20200540

**Published:** 2022-11-05

**Authors:** Ryosuke Fujii, Asahi Hishida, Takeshi Nishiyama, Masahiro Nakatochi, Keitaro Matsuo, Hidemi Ito, Yuichiro Nishida, Chisato Shimanoe, Yasuyuki Nakamura, Tanvir Chowdhury Turin, Sadao Suzuki, Miki Watanabe, Rie Ibusuki, Toshiro Takezaki, Haruo Mikami, Yohko Nakamura, Hiroaki Ikezaki, Masayuki Murata, Kiyonori Kuriki, Nagato Kuriyama, Daisuke Matsui, Kokichi Arisawa, Sakurako Katsuura-Kamano, Mineko Tsukamoto, Takashi Tamura, Yoko Kubo, Takaaki Kondo, Yukihide Momozawa, Michiaki Kubo, Kenji Takeuchi, Kenji Wakai

**Affiliations:** 1 Departments of Pathophysiological Laboratory Sciences, Nagoya University Graduate School of Medicine, Nagoya, Japan; 2 Department of Preventive Medical Sciences, Fujita Health University School of Medical Sciences, Aichi, Japan; 3 Department of Preventive Medicine, Nagoya University Graduate School of Medicine, Nagoya, Japan; 4 Department of Public Health, Nagoya City University Graduate School of Medical Sciences, Nagoya, Japan; 5 Public Health Informatics Unit, Department of Integrated Health Sciences, Nagoya University Graduate School of Medicine, Nagoya, Japan; 6 Division of Cancer Epidemiology and Prevention, Aichi Cancer Center, Nagoya, Japan; 7 Department of Cancer Epidemiology, Nagoya University Graduate School of Medicine, Nagoya, Japan; 8 Division of Cancer Information and Control, Aichi Cancer Center, Nagoya, Japan; 9 Department of Preventive Medicine, Faculty of Medicine, Saga University, Saga, Japan; 10 Department of Health Science, Shiga University of Medical Science, Otsu, Japan; 11 Department of Family Medicine, Cumming School of Medicine, University of Calgary, AB, Canada; 12 Department of International Island and Community Medicine, Kagoshima University Graduate School of Medical and Dental Sciences, Kagoshima, Japan; 13 Cancer Prevention Center, Chiba Cancer Center Research Institute, Chiba, Japan; 14 Department of Geriatric Medicine, Graduate School of Medical Sciences, Kyushu University, Fukuoka, Japan; 15 Laboratory of Public Health, School of Food and Nutritional Sciences, University of Shizuoka, Shizuoka, Japan; 16 Department of Epidemiology for Community Health and Medicine, Kyoto Prefectural University of Medicine, Kyoto, Japan; 17 Department of Preventive Medicine, Institute of Health Biosciences, the University of Tokushima Graduate School, Tokushima, Japan; 18 RIKEN Center for Integrative Medical Sciences, Yokohama, Japan

**Keywords:** hs-CRP, eGFR, Mendelian randomization study, genetic epidemiology, inflammation

## Abstract

**Background:**

Inflammation is thought to be a risk factor for kidney disease. However, whether inflammatory status is either a cause or an outcome of chronic kidney disease remains controversial. We aimed to investigate the causal relationship between high-sensitivity C-reactive protein (hs-CRP) and estimated glomerular filtration rate (eGFR) using Mendelian randomization (MR) approaches.

**Methods:**

A total of 10,521 participants of the Japan Multi-institutional Collaborative Cohort Study was analyzed in this study. We used two-sample MR approaches (the inverse-variance weighted (IVW), the weighted median (WM), and the MR-Egger method) to estimate the effect of genetically determined hs-CRP on kidney function. We selected four and three hs-CRP associated single nucleotide polymorphisms (SNPs) as two instrumental variables (IV): IV_CRP_ and IV_Asian_, based on SNPs previously identified in European and Asian populations. IV_CRP_ and IV_Asian_ explained 3.4% and 3.9% of the variation in hs-CRP, respectively.

**Results:**

Using the IV_CRP_, genetically determined hs-CRP was not significantly associated with eGFR in the IVW and the WM methods (estimate per 1 unit increase in ln(hs-CRP), 0.000; 95% confidence interval [CI], −0.019 to 0.020 and −0.003; 95% CI, −0.019 to 0.014, respectively). For IV_Asian_, we found similar results using the IVW and the WM methods (estimate, 0.005; 95% CI, −0.020 to 0.010 and −0.004; 95% CI, −0.020 to 0.012, respectively). The MR-Egger method also showed no causal relationships between hs-CRP and eGFR (IV_CRP_: −0.008; 95% CI, −0.058 to 0.042; IV_Asian_: 0.001; 95% CI, −0.036 to 0.036).

**Conclusion:**

Our two-sample MR analyses with different IVs did not support a causal effect of hs-CRP on eGFR.

## INTRODUCTION

Systemic inflammation is considered as one of the risk factors for common chronic diseases, including diabetes mellitus,^1^ hypertension,^2^ cardiovascular diseases,^3^ and chronic kidney disease (CKD).^4^ Generally, C-reactive protein (CRP) has been used as a biomarker of systemic inflammation in clinical and basic researches. Although the previous longitudinal studies have examined the association between CRP levels and CKD in different populations, evidence on the causality of this association remains controversial.^5^^–^^7^ However, some researchers demonstrated the effect of CRP-oriented biological functions on kidney function.^8^^,^^9^ One researcher also has published a meta-analysis suggesting that vitamin D supplementations could lower circulating CRP levels.^10^ Taken together, these studies suggest that interventions on CRP may help to improve renal function.

In the last few years, the Mendelian randomization (MR) approach has attracted much attention in genetic epidemiology. The biggest advantage of this method is to investigate a causal relationship between an exposure (X) and an outcome (Y) from an observational dataset using genetic variants as instrumental variables (G: IV).^11^ The development of MR analysis consecutively occurred after identifying single nucleotide polymorphisms (SNP) in genome wide association studies (GWAS). As with other health outcomes, previous GWAS identified SNPs associated with CRP levels, including the *CRP* gene in chromosome 1.^12^^,^^13^ Interestingly, it is known that serum CRP levels are affected by genetic polymorphisms,^14^ which indicates that SNPs associated with CRP levels may reflect the long-time exposure to higher/lower CRP level. Therefore, SNPs which associated with serum CRP levels are suitable for IVs to investigate the causal relationships between CRP and several pathophysiological conditions, and used in previous MR studies among adults in European countries.^15^^–^^17^

In Asian countries, large-scale cohort studies have collected human genome and performed genotyping in the past several decades. Several researchers conducted GWAS and found novel loci associated with CRP levels in Asian populations.^18^^–^^20^ These studies enable researchers to conduct MR study using CRP-associated SNPs in Asian populations, which seems to be important in terms of ethnic difference. Therefore, we investigated whether genetically determined hs-CRP levels using two different IVs, based on SNPs identified in European and Asian populations, were causally related to kidney function in a Japanese population using MR approaches.

## METHODS

### Study subjects

The study subjects were participants of the Japan Multi-institutional Collaborative Cohort (J-MICC) Study which was conducted in 14 study areas throughout Japan. The purpose of the J-MICC Study was to find out the risk factor of cancer and other diseases by examining the relationship between genetic variants, lifestyle habits, blood components, and disease. The eligibility for the J-MICC Study was adults aged 35–69 years living in each study area. The details of the J-MICC Study have been described previously elsewhere and the latest information is available on its website (http://www.jmicc.com).^21^^,^^22^ The selection process of participants is shown in eFigure 1. From the genotyped 14,539 subjects, 26 samples with inconsistent sex information between questionnaire and an estimate from genotype were excluded. The identity-by-descent method implemented in the PLINK 1.9 software (https://www.cog-genomics.org/plink2) identified 388 relative pairs (pi-hat >0.1875) and one sample of each pair was excluded. Principal component analysis with a 1,000 Genomes reference panel (phase 3) (http://www.internationalgenome.org/category/phase-3/) detected 34 subjects whose estimated ancestries were outlier from the Japanese population. The 34 samples were excluded. Among all the remaining 14,091 samples, five subjects withdrew their consent to participate, leaving 14,086 subjects for the final analyses. Of which, the values of serum hs-CRP were available only at three study sites. Therefore, we decided to use two-sample MR study design, rather than single sample MR for a smaller dataset. We divided the participants into two groups; 1) 2,503 participants (available for hs-CRP) and 2) 12,501 participants (non-overlapping participants), which are in accordance with a basic principle of two-sample MR (non-overlapping populations with same ethnicity, similar sex and age distribution).^23^ After excluding participants who had an extremely high value for hs-CRP (hs-CRP >3.0 mg/dL, *n* = 828) and eGFR (eGFR >120 mL/min/1.73 m^2^, *n* = 3,647), and lower than the limit of quantification for hs-CRP (*n* = 8), a total of 10,521 Japanese (1,667 for genetic association with hs-CRP [called as CRP dataset] and 8,854 for genetic association with eGFR [called as eGFR dataset]) were analyzed in the two-sample MR of this study. Written informed consent was obtained from all participants of this study. The J-MICC Study was conducted with adherence to the Ethical guidelines for the Human Genome and Genetic Sequencing Research. The procedure of this study was approved by the Ethics Review Committee of the Nagoya University Graduate School of Medicine (939-14), Aichi Cancer Center and all research institutes. We performed analyses using the dataset of version 20190728.

### Measurement of hs-CRP and eGFR

Serum samples were collected from all participants. We measured hs-CRP using a latex-enhanced nephelometry. Serum creatinine was basically measured using an enzymatic method. Some institutes measured serum creatinine using the Jaffe method, and then transformed to the equivalent value of the enzymatic method. eGFR was calculated using the Japanese equation proposed by the Japanese Society of Nephrology: eGFR (mL/min/1.73 m^2^) = 194 × serum creatinine (mg/dL)^−1.094^ × age^−0.287^ (× 0.739 for women).^24^

### Selection of instrumental variables

The list of candidate SNPs for IVs is shown in eTable 1. First, we selected four SNPs (rs3093077, rs1205, rs1130864, and rs1800947) within the *CRP* gene which was used as IVs in previous MR studies.^15^ These SNPs were selected as a minimum subset to obtain diversity at the *CRP* gene in European populations and called IV_CRP_ in this study. Next, we considered that it is necessary to select SNPs and develop original IVs in an Asian population because the IV_CRP_ was developed based on SNPs identified in people of European descents. Therefore, we searched the word of ‘CRP’ in the GWAS catalog (https://www.ebi.ac.uk/gwas/), and narrowed down to the studies according to the following criteria: 1) a study conducted in an Asian population, and 2) a study with both of the discovery and the replication phase. After the web-based selection, we finally selected 13 SNPs. For 151233628, due to low imputation quality (MAF <0.05 and *r*^2^ < 0.3), this SNP was not included in the original J-MICC dataset. Of remaining 12 SNPs, 6 SNPs (rs12133641, rs9375813, rs2097677, rs79802086, rs2393791, and rs1169284) were excluded because these SNPs were not significantly associated with hs-CRP in our dataset (*P* > 0.0042 = 0.05/12). Next, rs814295 (*GCKR*) and rs429358 (*APOE*) were likely to have pleiotropic effects on kidney function. rs3093059 was excluded due to the high linkage disequilibrium (LD) with rs3093068 in CRP dataset (*r*^2^ > 0.9). Finally, three SNPs (rs30933068, rs7553007, and rs7310409) were included in our analysis, and were called as IV_Asian_ (Table 2).

### Statistical analysis

To confirm the cross-sectional association between hs-CRP and eGFR, multiple linear regression analysis was performed with adjustment for sex, age, and study sites. We performed two-sample MR approaches after dividing the participants into two datasets (CRP and eGFR datasets) as described above. Methods for two-sample MR were different from one-sample MR method, which was described in previous methodological papers.^25^^–^^27^ The inverse-variance weighted method (IVW) is a conventional approach to estimate a causal effect on a study outcome from different studies in meta-analysis.^25^ In the setting of MR analysis, the IVW method can provide a combined estimate weighted using the inverse variances of the causal effect of per-allele. However, this method can be biased when a genetic variant violates the assumptions of MR (eg, pleiotropic effect).^27^ Therefore, we also performed two other methods (the weighted median (WM) and the MR-Egger method) which can provide consistent estimates even under the weaker assumption.^25^ The MR-Egger analysis is also useful to detect either/both directional pleiotropy or/and violation of the Instrument Strength Independent of Direct Effect (InSIDE) assumption. Additionally, the F-statistic was calculated for each IV from linear regression analyses to test whether IVs are strongly associated with exposure (referred to as relevance assumption).^28^ We performed linear regression analyses using *lm* function in R and included all SNPs used in each IV in models. An arbitrary threshold of F-statistic >10 was used to avoid using weak genetic instruments in this study.^29^ All statistical analyses were performed using the software R version 3.5.0 (R Foundation for Statistical Computing, Vienna, Austria). In particular, a R package of “MendelianRandomization” was used for two-sample MR analyses.^30^

## RESULTS

Table 1 shows basic characteristics of CRP and eGFR datasets. Mean ages of participants were not significantly different between the CRP dataset (55.5; standard deviation [SD], 9.6) and the eGFR dataset (55.1; SD, 9.2), and almost half of subjects were women in both datasets (CRP: 64.7% and eGFR: 53.4%). Median and interquartile range [IQR] of hs-CRP levels and eGFR was 0.04 mg/dL (IQR, 0.02–0.08) and 77.2 mL/min/1.73 m^2^ (IQR, 68.7–86.7).

**Table 1.** Demographic characteristics of participants in the hs-CRP and the eGFR dataset

	dataset (hs-CRP)	dataset (eGFR)
*n*	1,637	8,854
Age, years, mean (SD)	55.5 (9.6)	55.1 (9.2)
Female, *n* (%)	1,078 (64.7%)	4,726 (53.4%)
hs-CRP, mg/dL^a^	0.04 [0.02, 0.08]	—
eGFR, mL/min/1.73 m^2 a^	—	77.2 [68.7, 86.7]

hs-CRP, high sensitive C-reactive protein; eGFR, estimated glomerular filtration rate; SD, standard deviation.

^a^Values are expressed as median and interquartile range.

### Associations between instrumental variables and baseline hs-CRP

Two SNPs (rs3093077 and rs1205) in IV_CRP_ were significantly associated with ln(hs-CRP), but not for the other two SNPs (rs1130864 and rs1800947) (Table 2). All three SNPs in IV_Asian_ were associated with ln(hs-CRP). Combining these SNPs, both of IV_CRP_ and IV_Asian_ had a F-statistic >10 (14.8 and 22.5, respectively), which indicated that two IVs met a criterion for relevance assumption. Four SNPs in IV_CRP_ and three SNPs in IV_Asian_ explained 3.4% and 3.9% of the variation in hs-CRP, respectively.

**Table 2.** The SNP list for the two different instrumental variables (IV_CRP_ and IV_Asian_)

	SNP	Chromosome	Position^a^	EA^b^	ALT	EAF	Positional candidate gene	Estimate (SE)^c,d^	*P*-value^d^
IV_CRP_	rs3093077	1	159679636	*C*	*A*	0.146	*CRP*	0.389 (0.050)	4.25 × 10^−15^
	rs1205	1	159682233	*C*	*T*	0.328	*CRP*	0.213 (0.038)	1.97 × 10^−8^
	rs1130864	1	159683091	*A*	*G*	0.064	*CRP*	0.000 (0.075)	0.99
	rs1800947	1	159683438	*C*	*G*	0.979	*CRP*	0.205 (0.124)	0.10
IV_Asian_	rs3093068	1	159681364	*C*	*G*	0.142	*CRP*	0.391 (0.050)	5.51 × 10^−15^
	rs7553007	1	159698549	*G*	*A*	0.491	*CRP*	0.213 (0.038)	1.97 × 10^−8^
	rs7310409	12	121424861	*G*	*A*	0.525	*HNF1A*	0.128 (0.035)	3.01 × 10^−4^

ALT, alternative allele; BP, base pair; EA, effect allele; EAF, effect allele frequency; IV, instrumental variables; SE, standard error; SNP, single nucleotide polymorphism.

^a^The column of Position is presented base pairs based on Genome Reference Consortium Human Build 37 (GRCh37).

^b^The column of EA shows CRP increasing alleles.

^c^The estimates indicate a change of ln(eGFR) with an 1-unit increase in ln(hs-CRP).

^d^*P*-values was estimated by linear regression (R, ‘glm’) with adjustment for sex, age, and study sites.

### Conventional analysis for the association between hs-CRP and eGFR

Before the MR analysis, we performed the conventional statistical analysis for the cross-sectional association between hs-CRP and eGFR. Among 1,598 participants who were available on both of hs-CRP and eGFR, ln(hs-CRP) was strongly associated with ln(eGFR) (β = −0.015; 95% confidence interval [CI], −0.024 to −0.007; *P* = 3.26 × 10^−4^), after adjustment for sex, age, and study sites. The scatter plot for the association between ln(hs-CRP) and ln(eGFR) is shown in eFigure 2.

### Two-sample MR analysis

**Figure 1.**
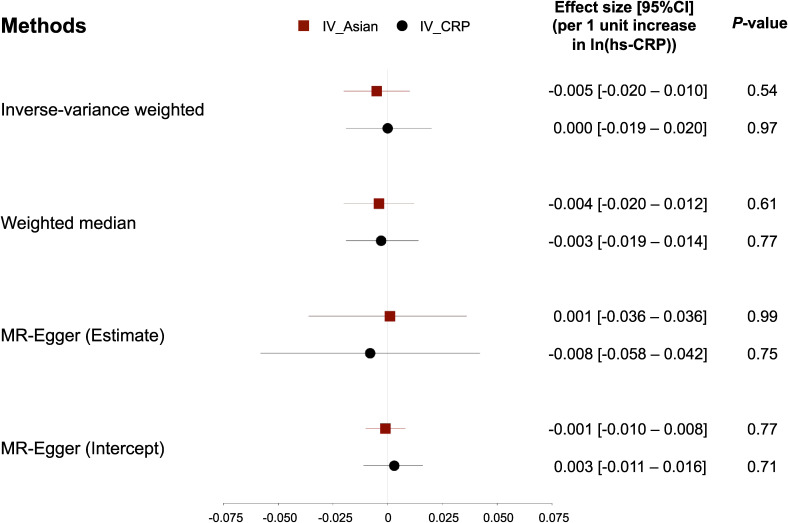
The results of two-sample MR study using two different IVs (IV_CRP_ and IV_Asian_). Effect sizes on the causal relationship between hs-CRP (X) and eGFR (Y) were estimated in three different MR methods. Estimates on eGFR are shown per 1 unit increase of log(hs-CRP). Red blocks and solid lines indicate the estimates and 95% CI using IV_Asian_, while black blocks and solid lines indicate the estimates and 95% CI using IV_CRP_. CI, confidence interval; eGFR, estimated glomerular filtration rate; hs-CRP, high-sensitivity C-reactive protein; IV, instrumental variable; MR, Mendelian randomization.

Using the IV_CRP_, genetically determined hs-CRP was not significantly associated with eGFR in the IVW method (estimate per 1 unit increase in ln(hs-CRP) = 0.000; 95% CI, −0.019 to 0.020; *P* = 0.97) (Figure 1, black blocks). Consistent with the result in the IVW method, no causal relationship was found in the WM method (estimate per 1 unit increase in ln(hs-CRP) = −0.003; 95% CI, −0.019 to 0.014, *P* = 0.77) and the MR-Egger method (estimate per 1 unit increase in ln(hs-CRP) = −0.008; 95% CI, −0.058 to 0.042; *P* = 0.75). The intercept estimated in the MR-Egger method was likely to be zero (estimate, 0.003; 95% CI, −0.011 to 0.016; *P* = 0.71). The scatter plot using IV_CRP_ is provided as eFigure 3A.

The estimated causal relationship using the genetic instruments reported in Asian populations (IV_Asian_) was not significant, which was consistent with the result using IV_CRP_ (Figure 1, red blocks). Estimates of ln(eGFR) per 1 unit increment in genetically determined ln(hs-CRP) in the IVW and the WM method were −0.005 (95% CI, −0.020 to 0.010; *P* = 0.54) and −0.004 (95% CI, −0.020 to 0.012; *P* = 0.61), respectively. The result in the MR-Egger was directionally inconsistent with two other methods, but still insignificant (estimate, 0.001; 95% CI, −0.036 to 0.036; *P* = 0.99). The intercept in the MR-Egger method was equal to zero (estimate, −0.001; 95% CI, −0.010 to 0.008; *P* = 0.77). The scatter plot using IV_Asian_ is provided as eFigure 3B.

## DISCUSSION

We assessed causality between genetically-determined inflammation and kidney function employing MR approaches in a Japanese population. In this study, we used four and three SNPs as different genetic instruments (IV_CRP_ and IV_Asian_). Neither of the two instrumental variables for hs-CRP was associated with eGFR levels in two-sample MR analysis. These results suggested no significant causal relationship between hsCRP and eGFR in this population.

We found that IV*_CRP_* was not significantly associated with eGFR, which indicates no causal relationship between genetically determined inflammation and kidney function. In this study, we used four SNPs (rs3093077, rs1205, rs1130864, and rs1800947) within the *CRP* gene as instrumental variables. A previous study reported that these four SNPs were selected as a set of tagging SNPs in the *CRP* gene.^17^ One of the previous MR studies in Caucasian reported that genetically determined CRP was not significantly associated with creatinine-based eGFR (β = 0.004; 95% CI, −0.01 to 0.02). Interestingly, this previous study used the same SNP set (IV_CRP_) as in this study, and the effect size of IV on eGFR was similar to that observed in the present study. Therefore, this insignificant association between genetically determined CRP level and eGFR is likely to be consistent across different ethnic groups.

The SNPs in IV_CRP_ were originally selected among individuals of European descent. Moreover, it is well known that the CRP level in Asian population was lower than that of Caucasians.^31^ Therefore, we tried to develop the IV specific for Asian people and selected three CRP-associated SNPs (rs3093068, rs7553007, and rs7310409) found in previous GWAS in Asian populations.^18^^–^^20^ However, no evidence found that IV_Asian_ was associated with eGFR in the study population.

IV requires the following three key assumptions: 1) relevance assumption (IV is associated with exposure), 2) exclusion restriction assumption (IV affects the outcome only through the exposure), and 3) exchangeability assumption (the effect of outcome is not confounded). Regarding relevance assumption, in this study, we restricted to only three SNPs in the robust selection process, thereby F-statistics of IV_Asian_ was relatively small (F-statistic = 22.5). Although this value barely satisfied the assumption of IV (F-statistic >10), the results were likely to be empirically verifiable for relevance assumption. Considering that we adopted the top significantly associated SNPs as IVs of CRP, which was not associated with renal functions, the contributions of genetically determined CRP on the risk of renal disease may be relatively limited compared to the multiple risk factors of this complex disease.^32^^,^^33^

Another key assumption for MR analysis is exclusion restriction assumption.^34^ This methodological review provided multiple scenarios violating this assumption (eg, inadequate phenotype definition and time-varying exposure). For scenarios of inadequate phenotype definition and measurement error, we used hs-CRP levels as an exposure variable. This is a clear definition of exposure and can lead to less measurement error compared with questionnaire-based phenotyping. For a scenario for the presence of LD, we carefully excluded either one of the SNPs in LD, which seems to have adequately addressed the problem. The scenarios of time-varying exposure and reverse causality are closely related and is usually worrisome for retrospective case-control study, where data on the exposure is collected after diagnosis of the outcomes. As above, we adequately addressed these problems, or our analysis has no substantial possibility to meet these scenarios. In addition to these scenarios, horizontal pleiotropy can violate exclusion restriction assumption. IV_Asian_ consisted of SNPs not only in *CRP* gene, but in *HNF1A*. Although previous studies suggested the SNP in *HNF1A* may have other pleiotropies,^35^^–^^37^ sensitivity analyses (MR-Egger and WM methods) in this study indicate that the IVW estimate was not biased by the average horizontal pleiotropic effect (known as directional pleiotropy).

The main strength of this study is that we used the IV specific for Asian populations. Given that CRP level is different in each population, the construction of IV_Asian_ might provide a meaningful approach of causal inference in Asian population. The present study also has limitations to be discussed. First, we created IV_Asian_ in this study, but investigated the association only in a Japanese population. Therefore, it remains unclear whether this result is consistent across Asian groups. Further studies are needed to examine this association in other Asian populations. Second, the number of SNPs used in IVs was small. Both of the two IVs met relevance assumption of IV in MR but were relatively weak. Given the relatively low variance of blood CRP levels and existence of a number of other determinants of CRP in human environments, such as bacterial infections or other inflammatory diseases not derived from inherited CRP levels, the contribution of genetically determined blood CRP levels may be limited in the development of human renal disease. Potentially, the bigger the number of SNPs in IV, the bigger the explained variance of the exposure. On the contrary, with an increment of SNPs in IV, the pleiotropic effects will also increase. In a recent paper, a researcher suggested that there was no need to exclude SNPs with pleiotropic effects.^37^^,^^38^ In this study, however, we prioritized selecting SNPs within the *CRP* gene over explanatory rate (ie, the number of SNPs in IV). In the future study, selection of IV will be more important. Third, the study sample size was relatively large in Asian populations, but this sample size may lead to limited power for two-sample MR (eMaterials 1). Therefore, the result needs to be validated in a larger dataset. In addition, it is difficult to conclude no causality between hs-CRP and eGFR because the estimated coefficient in the conventional analysis was included in the confidence intervals of the MR methods.

In conclusion, the present MR analyses investigated the causal relationship between hs-CRP and kidney function. Our two-sample MR analyses with two different IVs did not support a causal effect of hs-CRP on eGFR in this population.

## SUPPLEMENTARY MATERIAL

eMaterials 1. *Post-hoc* power calculation for two-sample Mendelian randomization

eFigure 1. Selection process of two-sample Mendelian randomization study.

eFigure 2. Scatter plot for the association between log(hs-CRP) and log(eGFR).

eFigure 3. Scatter plot for the associations between genetic association with log(hs-CRP) [G-X] and genetic association with log(eGFR) [G-Y] using IV_CRP_ (**A**) and IV_Asian_ (**B**). Red, green, and blue lines indicate the estimates of the IVW, the WM, and the MR-Egger analysis, respectively.

eTable 1. The candidate SNP list for the two different instrumental variables (IV_CRP_ and IV_Asian_)
