# Supplementary material for: Assessing the Relationship Between High-sensitivity C-reactive Protein and Kidney Function Employing Mendelian Randomization in the Japanese Community-based J-MICC Study
Source: J Epidemiol. 2022 Nov 5;32(11):483–8. doi: 10.2188/jea.JE20200540 (PMC9551292; doi:10.2188/jea.JE20200540)
Supplement: Supplementary file 1 [file je-32-483-s001.pdf]

Assessing the Relationship between High-sensitivity C-reactive Protein and Kidney Function Employing Mendelian Randomization in the Japanese Community based J-MICC Study

Ryosuke Fujii,[1, 2*] Asahi Hishida,[3] Takeshi Nishiyama,[4] Masahiro Nakatochi,[5] Keitaro Matsuo,[6, 7] Hidemi Ito,[8] Yuichiro Nishida,[9] Chisato Shimanoe,[9] Yasuyuki Nakamura,[10] Tanvir Chowdhury Turin,[10, 11] Sadao Suzuki,[4] Miki Watanabe,[4] Rie Ibusuki,[12] Toshiro Takezaki,[12] Haruo Mikami,[13] Yohko Nakamura,[13] Hiroaki Ikezaki,[14] Masayuki Murata,[14] Kiyonori Kuriki,[15] Nagato Kuriyama,[16] Daisuke Matsui,[16] Kokichi Arisawa,[17] Sakurako Katsuura-Kamano,[17] Mineko Tsukamoto,[3] Takashi Tamura,[3] Yoko Kubo,[3] Takaaki Kondo,[1] Yukihide Momozawa,[18] Michiaki Kubo,[18] Kenji Takeuchi,[3] and Kenji Wakai[3]

for the J-MICC Study Group

- **eMaterials 1.** *Post-hoc* power calculation for two-sample Mendelian randomization
- **eFigure 1.** Selection process of two-sample Mendelian randomization study.
- **eFigure 2.** Scatter plot for the association between log(hs-CRP) and log(eGFR).
- **eFigure 3.** Scatter plot for the associations between genetic association with log(hs-CRP) [G-X] and genetic association with log(eGFR) [G-Y] using IV_{CRP} (**A**) and IV_{Asian} (**B**). Red, green, and blue lines indicate the estimates of the IVW, the WM, and the MR-Egger analysis, respectively.
- **eTable 1.** The candidate SNP list for the two different instrumental variables (IV_{CRP} and IV_{Asian})

eMaterials 1. *Post-hoc* power calculation for two-sample Mendelian randomization

We conducted power calculation for two-sample MR using R codes from a recent paper from Deng L, et al. *Genet Epidemiol.* (2020). For both IV_{CRP} and IV_{Asian}, we estimated power as follows:

Basic Information for input:

- the specified significance level (alpha): 0.05
- the variance of the risk exposure (X): 1.07
- the variance of the outcome (Y): 0.04
- the number of subjects having measure on (Y,Z)sample: 8,854
- the number of subjects having measure on (X,Z)sample: 1,667

IV_{CRP}

the proportion of the total variation of exposure X that can explained by IVs (Z): 0.03

true causal effect (beta)	estimated power
-0.020	0.238
-0.025	0.375
-0.030	0.525

IV_{Asian}

the proportion of the total variation of exposure X that can explained by IVs (Z): 0.04

true causal effect (beta)	estimated power
-0.020	0.475
-0.025	0.647
-0.030	0.787

Deng L, Zhang H, Yu K. Power calculation for the general two-sample Mendelian randomization analysis. Genet Epidemiol. 2020;44:290-299.

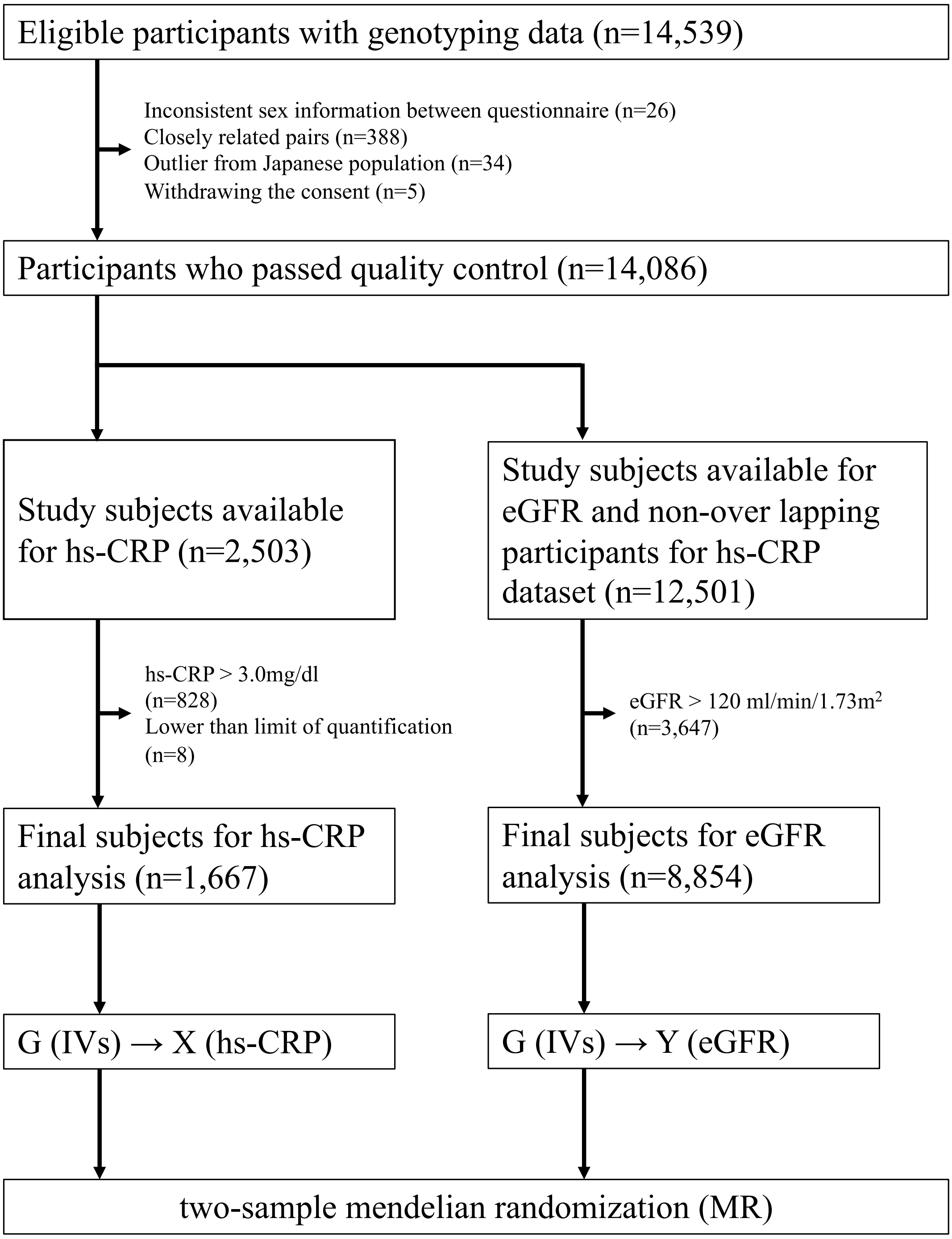

eFigure 1. Selection process of two-sample Mendelian randomization study

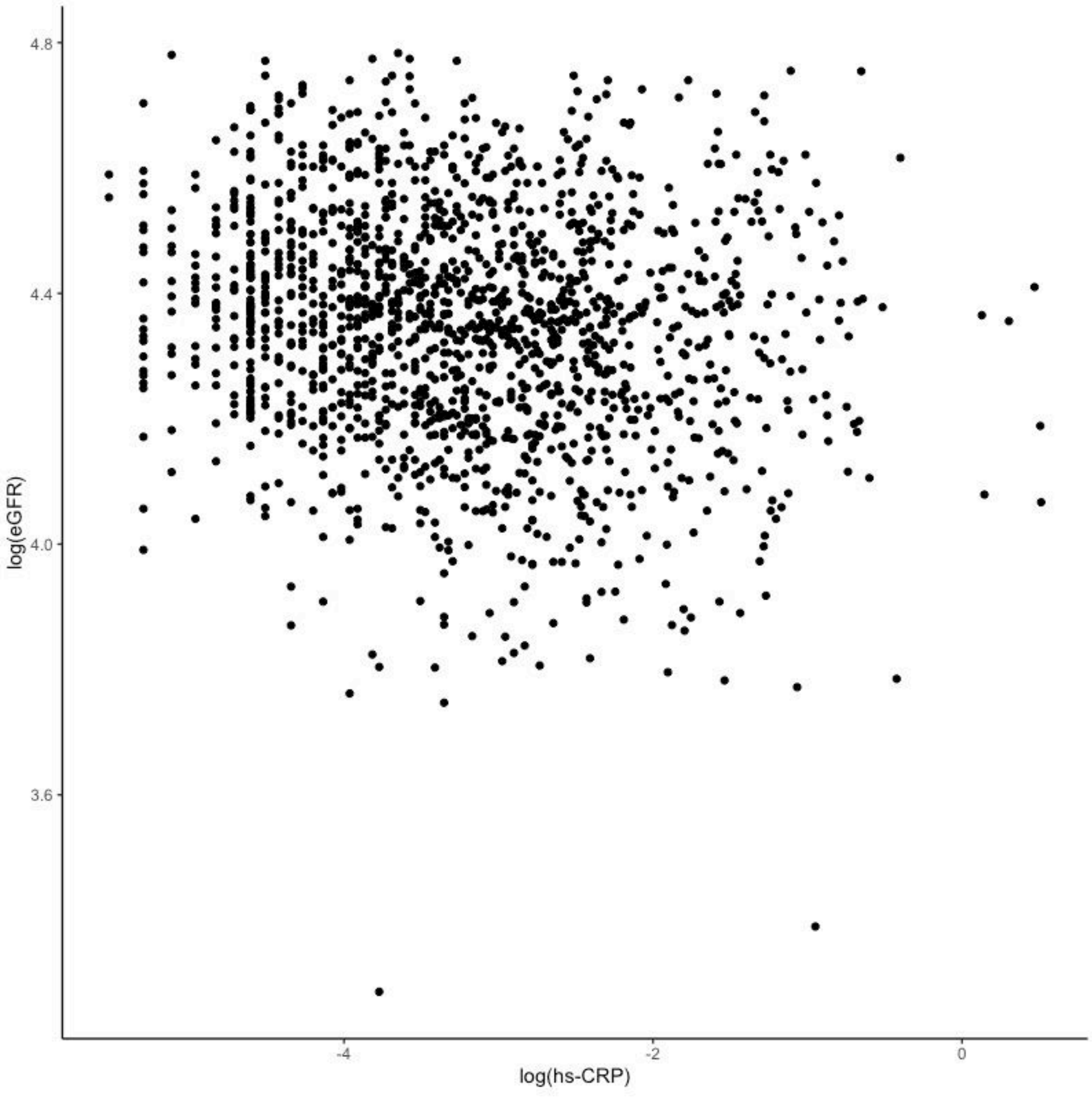

eFigure 2. Scatter plot for the association between log(hs-CRP) and log(eGFR)

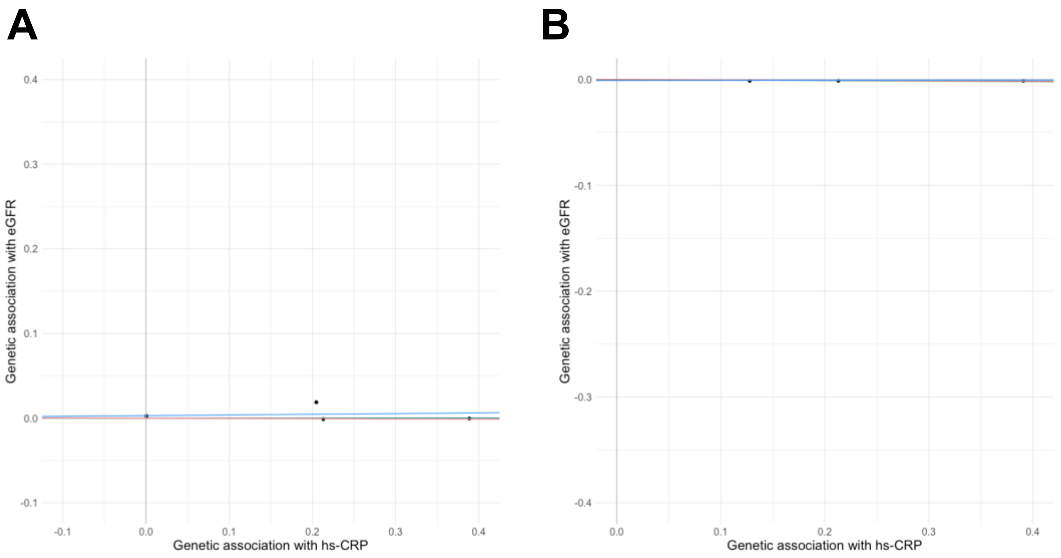

eFigure 3. Scatter plot for the associations between genetic association with log(hs-CRP) [G-X] and genetic association with log(eGFR) [G-Y] using IV_{CRP} (**A**) and IV_{Asian} (**B**). Red, green, and blue lines indicate the estimates of the IVW, the WM, and the MR-Egger analysis, respectively.

eTable 1. The candidate SNP list for the two different instrumental variables (IV_{CRP} and IV_{Asian})

	SNP	Chromosome	Position (BP)[a]	Allele[b,c]	Positional candidate gene	References	Reasons for exclusion
IV_{CRP}	**rs3093077**	**1**	**159679636**	***G***	***CRP***	**CRP CHD genetics collaboration. Eur J Epidemiol (2008)**	–
	**rs1205**	**1**	**159682233**	***C***	***CRP***	**CRP CHD genetics collaboration. Eur J Epidemiol (2008)**	–
	**rs1130864**	**1**	**159683091**	***T***	***CRP***	**CRP CHD genetics collaboration. Eur J Epidemiol (2008)**	–
	**rs1800947**	**1**	**159683438**	***G***	***CRP***	**CRP CHD genetics collaboration. Eur J Epidemiol (2008)**	–
IV_{Asian}	rs12133641	1	154428283	*G*	*IL6R*	Kanai M, et al. Nat Genet (2018)	Not significant
	**rs3093068**	**1**	**159681364**	***G***	***CRP***	**Kanai M, et al. Nat Genet (2018)**	**–**
	rs3093059	1	159685136	*G*	*CRP*	Okada Y, et al. Hum Mol Genet (2011)	LD with 3093068
	**rs7553007**	**1**	**159698549**	***G***	***CRP***	**Vinayagamoorthy N, et al. PLoS One (2014)**	**–**
	rs814295	2	27743215	*G*	*GCKR*	Kanai M, et al. Nat Genet (2018)	Pleiotropy
	rs9375813	6	131757247	*C*	*ARG1*	Vinayagamoorthy N, et al. PLoS One (2014)	Not significant
	rs2097677	7	22732839	*A*	*IL6*	Okada Y, et al. Hum Mol Genet (2011)	Not significant
	rs79802086	7	22735932	*T*	*LOC401312, LOC541472*	Kanai M, et al. Nat Genet (2018)	Not significant
	rs7310409	12	121424861	*G*	*HNF1A*	Okada Y, et al. Hum Mol Genet (2011)	Not significant
	rs2393791	12	121423956	*A*	*HNF1A*	Vinayagamoorthy N, et al. PLoS One (2014)	Not significant
	**rs7310409**	**12**	**121424861**	***G***	***HNF1A***	**Okada Y, et al. Hum Mol Genet (2011)**	**–**
	rs151233628	17	6161785	*C*	*WSCD1, AIPL1*	Kanai M, et al. Nat Genet (2018)	Low quality of imputation
	rs429358	19	45411941	*C*	*APOE*	Kanai M, et al. Nat Genet (2018)	Pleiotropy

BP, base pair; LD, linkage disequilibrium; IV, instrumental variable

[a]The column of position (BP) is presented based on Genome Reference Consortium Human Build 37 (GRCh37).

[b]The column of Allele shows CRP increasing alleles in the original study (not in this study).

[c]The SNPs used in our study was shown in bold letters.
